# QIR and SQUIRE: continuum of reporting guidelines for scholarly reports in healthcare improvement

**DOI:** 10.1136/qshc.2008.029074

**Published:** 2008-09-26

**Authors:** R G Thomson, F M Moss

**Affiliations:** 1 Institute of Health and Society, Newcastle Medical School, Newcastle upon Tyne, UK; 2 London Deanery, London, UK

Significant problems with the quality and safety of care seem endemic in all healthcare systems. In a recent systematic review of case note review studies, over 9% of patients admitted to hospital are harmed by error.^1^ Furthermore, many patients do not get the treatment that would be effective for them and many more have care that is inefficient.^2^ When any problem is investigated the solution may appear simple. However, getting necessary systematic change always seems difficult.

The quality improvement movement, and latterly the safety movement, came late to health care compared to other industries. Perhaps this is in part a reflection of the additional complexity of health care. While problems with care are not new, in the past 20 years or so there has been a huge increase in the number of effective treatments and in their complexity, with an increase in public concern about the safety and quality of care.^3^ There is much theory and practice to be learned from other industries. But theory alone is not enough and there is a clear imperative to find practical ways of addressing quality and safety in local healthcare settings in a robust and reliable way—we owe this both to our patients and to ourselves.

In 1991 we helped to found the journal now called *Quality and Safety in Health Care*. We sought to provide a forum for the exchange of scholarly ideas, original research, debate and discussion, and examples of good practice. The survival and growth of the journal attests to the importance of the field and those initial aims. Although there may now be an improved understanding among a few about what is wrong with health care and how it could be put right, the fact that systematic problems with care—for example, infection control in hospital (present seven years ago)—still exist in much the same way, suggests there is a long way to go in the practical application of quality improvement methods.

Finding out what works in the real world of clinical practice that could be of use to others facing the same problems and then disseminating this was one of the initial aims of *Quality and Safety in Health Care*. Initially we encouraged and published such reports. But we did not provide any specific guidance; we asked authors to use the standard IMRaD (Introduction, Methods, Results and Discussion) structure that is used for research papers and we received and published very few. Providing a platform for the dissemination of examples of good locally based quality improvement work was proving hard to achieve. Experience at conferences, where many examples of innovative, stimulating and even inspiring projects were being presented, suggested that much work was being done, but that little made it as far as publication. Our own local intelligence suggested that even more interesting and potentially useful work was not even being presented at conferences. This was undermining the potential for wider sharing and uptake of experiential learning.

Several reasons explained the sparse publication of quality improvement reports. First, leaders of such projects were rarely academics and did not have either the incentives or perhaps the skills and experience to write for publication. Second, what drove them, and where they would invest any extra time and energy, was not writing what they had done, but the challenge and rewards of further improvements to care. Third, there had been few journals interested in publishing “grassroot” quality improvement work. Finally, the nature of quality improvement work differs in a number of ways from research.

A fundamental difference between quality improvement reports (QIR) and the reports of original research is that research seeks broadly to produce generalisable results but quality improvement work seeks to test the application of those results. Thus, trials of thrombolytic treatment in acute myocardial infarction sought to determine whether thrombolysis reduced subsequent mortality, such that the results could be generalised to coronary care units and medical wards treating such patients and a general statement could be made that “all patients with myocardial infarction who meet defined criteria *should* get thrombolytic therapy”. A subsequent local audit or quality improvement project would seek to assess whether all eligible patients were appropriately treated with thrombolytic therapy. If problems were found, then local quality improvement would go on to define the local problems and then seek to change the system of care so that “all patients with myocardial infarction who meet defined criteria *do* get thrombolytic therapy”. The results of such a study may not be generalisable to other coronary care units in the same way as the preceding research evidence—they may be unique to the local context of the unit in which the audit was undertaken. None the less, a well written and structured quality improvement report may include generalisable methods and strategies for change from which others undertaking similar audits would benefit, thus disseminating good practice. The interest to others is as much in the details of strategies for change as in the outcome.

A problem faced by those intending to write about their quality improvement work was that the IMRaD structure, so helpful for exposing the essential elements of research, was unnecessarily constraining when writing about quality improvement projects. IMRaD did not reflect or fit the thinking of practical quality improvement and did not easily allow authors to describe the cyclical nature of their work. Few papers that used this structure to report quality improvement work managed to get to the heart of the matter—namely, to clearly describe strategies for change and the lessons for others.

To support those whom we knew were doing interesting and informative quality improvement work, we revised the structure for QIR and, in discussion with expert colleagues and the board of *Quality and Safety in Health Care*, we set out guidelines for their publication that, in particular, set out to address the perceived problems of applicability of the IMRaD structure. These have now been used by *Quality and Safety in Health Care* since 1999^4^ and the *BMJ* since 2000,^5^ and over 50 QIR have been published using this format. This structure has also been used for abstracts for the international and European forums on quality and the basis of writing workshops.

The approach adopted for developing the QIR structure was pragmatic and addressed our need as editors to encourage submission of locally based quality improvement work that made sense and was useful to others. Unlike the subsequent development of other publication guidelines such as CONSORT,^6^ or indeed the SQUIRE guidelines published in this supplement,^7^ we did not use an extensive consensus building approach. However, despite a less formal approach to development, the QIR structure for locally based quality improvement work seems to have met a need.

As the scientific basis of quality improvement and safety has developed, larger multicentre evaluations of quality improvement tools and methods have emerged. At the same time the evidence-based medicine movement became hugely influential, with the Cochrane Collaboration emphasising the superiority of randomised controlled trials in evaluating the effectiveness of interventions. Critics of the robustness and validity of quality improvement work became increasingly prominent and a polarising debate developed, perhaps best characterised by criticisms of the evaluation of the Institute for Healthcare Improvement 100 000 lives campaign.^8^ Core to this debate was a concern about attribution—how can we be sure that the interventions described led to the observed improvements? And, if we cannot be sure, how do we avoid making important policy decisions based on potentially flawed method, analysis and interpretation?

There was another distinction that we made when we first developed the QIR structure; this was the distinction between generalisable results that arise from robust empirical research into the effectiveness of interventions and generalisable methods and approaches (including experiential learning) that derive from reports of locally based quality improvement projects. QIR therefore provided a basis for sharing experience and learning rather than disseminating generalisable results. On the other hand, for those involved in seeking to undertake effective quality improvement work there is a clear need for robust research using appropriate methods to produce generalisable results demonstrating the effectiveness of quality improvement interventions.^9^^–^12

On the back of this perfectly reasonable challenge to undertake robust quality improvement research, Davidoff and Batalden suggested a new draft structure for publication of such research.^13^ As described in more detail in this supplement, this led on to the development of the SQUIRE guidelines, drawing upon learning from methods of development of other publication guidelines such as CONSORT^6^ and QUORUM.^14^

Now that we have the SQUIRE guidelines would or should they replace QIR guidelines? Or do they have a different purpose? Perhaps SQUIRE for research and QIR for describing locally based improvement initiatives? One of us (RT) actively engaged with the group constituted to develop the SQUIRE guidelines, while the other (FM) took a close interest from the sidelines and used the QIR structure for teaching and for abstracts describing quality improvement work. As the original architects of QIR, and in the spirit of continuous improvement, we realised that the original QIR would benefit from review and updating, but we also thought that merit remained in the original formulation and were uncomfortable with the return to IMRaD. In order to meet the rigour and essential details of quality improvement research, the SQUIRE guidelines are much longer and necessarily more complex than the QIR structure and follow the IMRaD structure; therefore, they might put off those wanting to write up and share locally based, but effective, quality improvement projects and perhaps not encourage the reflective intent of the QIR guidelines.

So what is the way forward? Can one set of guidelines meet both needs? Can we benefit from the development of SQUIRE while retaining the strengths of QIR? How can publication guidelines support dissemination of both quality improvement projects and robust quality improvement research? Quality improvement work should of course be rigorous; indeed, there is much that could be done to enhance the quality and generalisability of such locally based work, not least the use of robust and valid measurement tools.^10^ And it is robust and generalisable quality improvement research that will provide the basis for understanding the effectiveness of such tools. It is crucial that the effectiveness and utility of all methods are understood so that those doing local work can know which tools to use, in which circumstances and how to use them. On the other hand, the combination of different methods, both quantitative and qualitative, done well, can enhance the capacity to demonstrate attribution—through triangulation of results—and support dissemination of experiential learning when randomised controlled trials or more robust evaluation methods are inappropriate, unethical or impractical, as is often the case in local settings.^10^ ^11^

We would argue that there is a spectrum of activity ranging from local uncontrolled quality improvement projects to robust multicentre studies evaluating quality improvement methods and interventions. Between these extremes lie studies that use mixed methods or less robust approaches. Furthermore, the amount of (informal but useful) experience and learning is probably considerably in excess of the body of planned experimental evaluative studies in quality improvement. The SQUIRE guidelines will be a hugely significant development to support not only the reporting, but also the design and undertaking, of robust quality improvement research into the effectiveness of quality improvement interventions. However, they have limitations in supporting the writing up and publication of smaller local projects; we would argue that this is where QIR guidelines retain their value. Towards the centre of the spectrum there may well be a choice between QIR and SQUIRE that could be driven by the form and detail of the evaluation undertaken or perhaps by author (or journal) preference.

If this argument is accepted, then further work is needed. First, it is time QIR structure and guidance were reviewed. This can be informed by several years of experience of QIR application, as well as by the SQUIRE conclusions, and perhaps at this stage should now include a more structured consensus approach. To kick this off, we invite comments from readers of this article (http://www.squire-statement.org). In addition we will approach authors of QIR to seek their views and will draw upon a recent, as yet unpublished, comprehensive review of QIR (Kate Kaplan, personal communication). None the less, any review of QIR would need to be cognisant of the fact that much of their apparent success in supporting the reporting and publication of experiential quality improvement projects lies in their apparent simplicity. While recognising that the SQUIRE guidelines are much more complex in order to cater for the additional complexities of robust research into quality improvement interventions, we would be wary of expanding the QIR guidance and risk turning off the still rather slowly running tap of publication of quality improvement reports.

Improving the quality and safety of care is of critical importance to all who work in health care. Much is known, but so far too little has been translated into effective change. We need both more research into quality improvement methods, as well as more information about what works locally. To support dissemination we need structures that will facilitate the writing and expression of important messages for others. Research and practical experience are both important contributors to our knowledge in this field. We need accessible guidance that meets the needs of authors and publishers of both.
